# DeepGeni: deep generalized interpretable autoencoder elucidates gut microbiota for better cancer immunotherapy

**DOI:** 10.1038/s41598-023-31210-w

**Published:** 2023-03-21

**Authors:** Min Oh, Liqing Zhang

**Affiliations:** 1 Department of Computer Science, Virginia Tech, Blacksburg, VA USA; 2 Microsoft Research, Redmond, WA USA

**Keywords:** Predictive markers, Machine learning, Clinical microbiology

## Abstract

Recent studies revealed that gut microbiota modulates the response to cancer immunotherapy and fecal microbiota transplantation has clinical benefits in melanoma patients during treatment. Understanding how microbiota affects individual responses is crucial for precision oncology. However, it is challenging to identify key microbial taxa with limited data as statistical and machine learning models often lose their generalizability. In this study, DeepGeni, a deep generalized interpretable autoencoder, is proposed to improve the generalizability and interpretability of microbiome profiles by augmenting data and by introducing interpretable links in the autoencoder. DeepGeni-based machine learning classifier outperforms state-of-the-art classifier in the microbiome-driven prediction of responsiveness of melanoma patients treated with immune checkpoint inhibitors. Moreover, the interpretable links of DeepGeni elucidate the most informative microbiota associated with cancer immunotherapy response. DeepGeni not only improves microbiome-driven prediction of immune checkpoint inhibitor responsiveness but also suggests potential microbial targets for fecal microbiota transplant or probiotics improving the outcome of cancer immunotherapy.

## Introduction

Recent studies have found that the composition of the gut microbiome modulates the response to cancer immunotherapies^1–3^. Immune checkpoint inhibitors (ICIs) that block immunosuppressive molecules of tumor cells, thereby inducing host immune response are highly effective for only a subset of patients (~ 40%)^4^. The gut microbiome has been reported as a major extrinsic modulator to responses of ICIs such as anti-PD-1. In mice, fecal microbiota transplantation (FMT) from responders to non-responders promotes the efficacy of anti-PD-1 therapy in non-responders^1–3^. More recently, first-in-human clinical trials observed the clinical benefit of responder-derived FMT in melanoma patients^5,6^. Although the gut microbiome is associated with response to anti-PD-1 therapy, its composition and the specific mechanisms affecting host immune response remain unclear^7^.

Determining the key microbiota affecting individual responses to cancer treatment is crucial for advancing precision oncology. However, this is challenging due to the limited available data sets, thereby a lack of generalizability in statistical and machine learning models. For example, multiple studies on small melanoma cohorts have reported gut bacteria associated with response to ICI therapy^1,2,8–10^, but unfortunately, there are discrepancies in the findings^7^. Many bacteria reported by those studies did not appear in multiple studies at the species level except *Faecalibacterium prausnitzii* and *Bacteroides thetaiotaomicron*. Also, previous attempt to train machine learning classifiers on microbiome profiles has shown relatively low accuracy in the prediction of ICI response on unseen data^11^. This suggests the need for the curation of massive-scale studies to obtain statistical power to generalize microbial signatures to unseen data.

Nevertheless, recent advances in artificial intelligence, especially deep learning models for domain generalization may hold promise in generalizing microbial signatures. Domain generalization, also called out-of-distribution generalization, aims at learning models that can be generalized to an unseen domain without any foreknowledge^12^. Domain generalization techniques usually require data from multiple domains or sufficient enough to simulate domain shifts, and the limited availability of microbiome data often restricts the application of the techniques. However, more recent studies proposed data augmentation approaches, circumventing the limitation^13–15^. Especially, DeepBioGen showed promise in augmenting limited sequencing data, including microbiome profiles, and improving the generalizability of classification models^16^.

Well-generalized and accurate deep learning models have the potential to be a key part of clinical decision-making in precision medicine^17,18^. Despite the remarkable performance, deep learning models are usually black-box and difficult to interpret, which hampers their adoption in clinical practice as clinicians and decision-makers prioritize the explainability of the predictions^19^. Also, interpretable models may provide insight into the underlying mechanisms connecting gut microbiome and host immune response.

**Figure 1:**
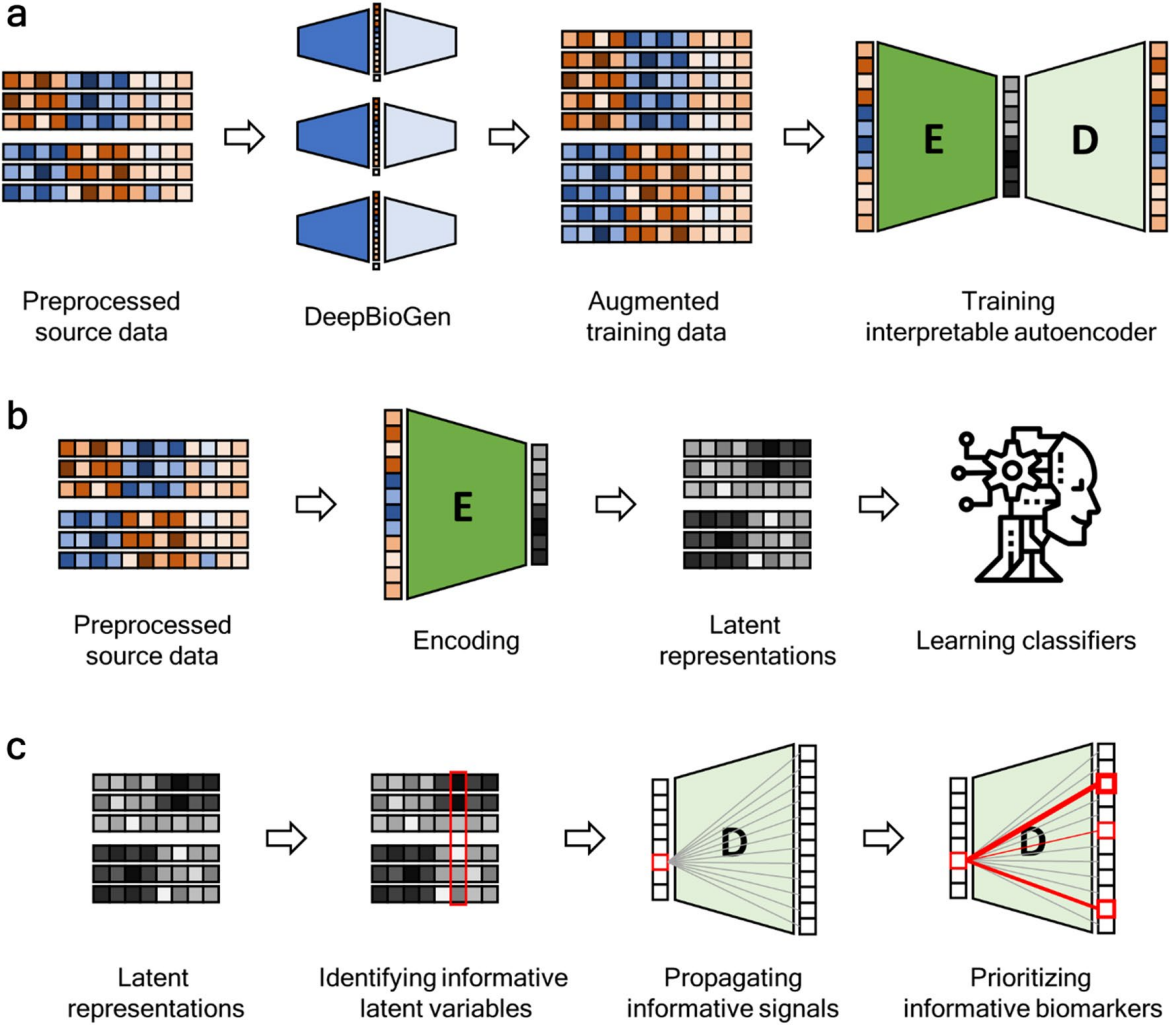
Overview of DeepGeni analysis. (**a**) The source microbiome profiles are augmented with realistic profiles generated from DeepBioGen and an interpretable autoencoder is trained on the augmented training data. (**b**) Through the encoder of the trained autoencoder, the source microbiome profiles are transformed into latent representations that are used to train classifiers predicting ICI responsiveness. (**c**) The informative latent variables are identified with a feature selection algorithm and their signals are propagated through weights on the decoder of the trained autoencoder to identify the informative microbial markers.

In this study, DeepGeni, a deep generalized interpretable autoencoder, is proposed to unveil the gut microbiome associated with ICI response (Fig. 1). A previous study has shown that a deep autoencoder can produce a highly effective representation of microbiome profiles^20^. Also, a flexible autoencoder model has been developed for interpretable autoencoding without a significant loss of reconstruction accuracy^21^. By augmenting microbiome profiles and by introducing explainable links in the autoencoder, DeepGeni improved not only the generalizability but also the interpretability of the learned representation of microbiome profiles. DeepGeni-based classifiers outperform a state-of-the-art classifier in predicting ICI response using microbiome profiles. Also, interpretable links of DeepGeni reveal important taxa for ICI response prediction, and the identified taxa are either associated with prolonged progression-free survival in melanoma patients treated with ICI therapy or differentially abundant between responders and non-responders. DeepGeni source code is free and available at https://github.com/minoh0201/DeepGeni.

## Methods

### Datasets

Gut microbiome data of melanoma patients treated with ICI therapy were collected from four shotgun metagenomic studies^1,2,9,22^. This study focused on samples gathered before ICI therapy and excluded the other samples taken after ICI administration. Patients’ responsiveness to ICI therapy was evaluated with RECIST 1.1 criteria where complete or partial responses are classified as responders and stable or progressive disease states as non-responders^23^. Since Peters et al.’s data did not have an explicit classification of responsiveness, patients with over 6 months of progression-free survival were regarded as responders and the others as non-responders as suggested by Limeta et al.^11^. In total, 130 melanoma patients (66 responders and 64 non-responders) were used (Table 1).

**Table 1:** Summary of gut microbiome datasets derived from shotgun metagenomic sequencing.

Dataset name	# Of total samples	# Of responders	# Of non-responders	Published year	ICI therapy	Reference
Gopalakrishnan	25	14	11	2018	Anti-PD-1	^1^
Matson	39	15	24	2018	Anti-PD-1	^2^
Frankel	39	19	20	2017	Anti-PD-1, Anti-CTLA-4, and both	^9^
Peters	27	18	9	2019	Anti-PD-1, Anti-CTLA-4, and both	^22^

Raw sequencing reads were filtered with FASTP and processed with mOTUs2, a phylogenetic (mOTU) profiler^24,25^. Processed microbiome profiles containing read counts for each phylogenetic marker gene and each patient were acquired from Limeta et al.^11^. Read counts were normalized by the total number of reads for each patient, and then log2-transformed. In total, 7,727 mOTUs (features) were considered in an initial input.

### Microbiome profile augmentation with DeepBioGen

DeepGeni utilizes DeepBioGen^16^, a sequencing profile augmentation procedure that generalizes the subsequent trainable models with the augmented data (Fig. 1a). Visual patterns of source microbiome profiles are established with feature selection followed by feature-wise clustering. Wasserstein generative adversarial network (GAN) equipped with convolutional layers capturing the visual patterns generates realistic profiles and augments source data. The augmented training data can enhance the generalizability of the subsequent models such as machine learning classifiers to unseen data. In this study, DeepBioGen parameters were set to default, otherwise, configured following the guideline described in the original paper. Test data has been excluded from any estimation of the parameters. Out of 7727 mOTU features, 256 features were selected by fitting extremely randomized trees on source data^26^. The number of feature-wise clusters and the number of GAN models were estimated by calculating the within-cluster sum of squared errors in source data with reduced features. To visualize augmented data along with source and test data in high-dimensional space, t-SNE algorithm^27^ was used to embed the data points in 2-D space (Fig. S1). The number of iterations and perplexity were set to 1000 and 50, respectively. Scikit-learn package (version 0.22.2) was used to run the implementations of extremely randomized trees, the k-means clustering algorithm for calculating the within-cluster sum of squared errors, and the t-SNE algorithm. The final release of DeepBioGen was forked (April 2021) from the repository provided by authors (https://github.com/minoh0201/DeepBioGen/) and executed on a docker image running Tensorflow 1.13.2 as instructed.

### Generalized autoencoder with interpretable links

Autoencoder consists of encoder and decoder functions that are approximated by neural networks. The encoder maps the input data points into latent space and the decoder reconstructs the input from the mapped latent representations. During training, the autoencoder tries to minimize the gap between the input and the reconstruction by adjusting the weights of neural networks based on back-propagated signals from the reconstruction loss term. Formally, the reconstruction loss can be written as,

$$L\left( {x,x^{^{\prime}} } \right) = x - x^{^{\prime}2} = x - f_{\theta }^{^{\prime}} \left( {f_{\phi } \left( x \right)} \right)^{2},$$

where $x$ and $x^{^{\prime}}$ are the input and the reconstruction, $f_{\phi } \left( \cdot \right)$ and $f_{\theta }^{^{\prime}} \left( \cdot \right)$ are encoder and decoder functions in which $\phi$ and $\theta$ are their weights, respectively. The latent representation usually has a smaller dimension than the original input but it contains concentrated information that can be used to reconstruct the original input with minimal error. Although the latent representation may hold essential information in a condensed form, it is not directly interpretable because of the non-linear relationship between latent and original features.

Svensson et al. suggested a flexible autoencoder model removing non-linearity in the decoder function, opening up the possibility to retain interpretability without ruining reconstruction quality^21^. The non-linearity of the autoencoder comes from a non-linear activation function applied to the weighted sum of the preceding inputs. By removing the activation function in the decoder part, direct linear links from the latent layer to the output layer can be obtained. In this study, simple autoencoder architectures composed of three dense layers were utilized: input layer, latent layer, and output layer. The number of nodes of the input and output layers is the same as that of the input. Four different sizes of latent nodes were examined: 128, 64, 32, and 16. The augmented training data consisting of source and augmented data was used to train the autoencoder. After training, the encoder part was used to produce latent representations of the augmented training data. Test data was isolated from any steps of autoencoder training. We used Tensorflow (version 1.13.2) and Keras (version 2.3.1) libraries to implement the interpretable autoencoder.

### Generalized latent representations for predicting ICI responses

To estimate the usefulness of the latent representations derived from the generalized autoencoder, prediction models classifying ICI responses were built on the representations (Fig. 1b). Three machine learning algorithms, support vector machine (SVM), random forest (RF), and multi-layer feedforward neural network (NN) were used to train the models (implemented using Scikit-learn 0.22.2). Prediction performance was evaluated with two approaches. The first approach, similar to Limeta et al., utilizes the most recent dataset in Peters et al. (Peters) as test data^22^, and the remaining data pooled together as source data. The other approach is cross-study validation which iterates over datasets, leaves one dataset as test data, uses the remaining as source data, and averages over results. For both approaches, five-fold cross-validation on the learned representation of source data was conducted to optimize the hyperparameters of the classification algorithms. Hyper-parameter space was explored with grid search and the parameter grid is described in Supplementary Table S1. With the best hyper-parameters, classifiers were trained on representations of the entire source data and evaluated on test data. The area under the receiver operating characteristics curve (AUC) was used to assess the prediction performance.

### Extracting informative microbiota from interpretable autoencoder

To interpret the latent representations that improve the prediction of ICI response, the most informative latent variables were selected based on feature importance estimated by extremely randomized trees^26^. The informative signals of the selected latent variables were propagated through direct links in the decoder network (Fig. 1c). Out of 128 latent variables, ten of the most informative variables were considered for further analysis. For each variable, the links were ranked by the absolute value of their weights, and, out of 256 links, the top 20 were selected. After the corresponding output nodes connected to the top 20 links were mapped to mOTUs in a one-to-one manner, the specified 20 mOTUs were listed in a set of candidates. By iterating over the ten latent variables, the ten sets of candidates were merged into a unique set of candidates. The whole process was repeated four times by dropping one data set at a time and using the rest for better generalizability. From four supersets, each of which had different 256 features (Fig. S2), four candidate sets were derived. Each of the four subsets had 140, 139, 144, and 141 candidates respectively. The finalist was acquired by taking the intersection of the four sets of candidates and it contains 14 mOTUs (permutation testing *p*-value = 9.0 × 10^–6^).

### Statistical analysis

The statistical significance of the informative microbiota extracted by taking the intersection of four sets of candidates was assessed using permutation testing (n = 1,000,000, *p* < 0.01). We counted the number of permutations whose number of intersecting microbiota is greater than or equal to that of the finalist and obtained a p-value estimating the random chance of getting such an intersection.

To assess the impact of the identified informative mOTUs on ICI responsiveness, progression-free survival analysis which is a primary endpoint of clinical oncology studies was conducted. Data in Peters et al. (N = 27) has progression-free survival and was used in the analysis. For each mOTU, the second quartile (median) was used as a cut-off for high abundance. The Kaplan–Meier plot was drawn and the log-rank test was conducted for statistical significance. Wilcoxon rank-sum test was used to determine differentially abundant taxa.

## Results

### Improved prediction of ICI response with generalized interpretable autoencoder

We evaluated the prediction performance of machine learning classifiers utilizing DeepGeni, a deep generalized interpretable autoencoder. The classifiers were learned to predict a binary class of ICI treatment (responder vs non-responder) based on the latent representation of microbiome profiles. Test data has been excluded from the whole process of generalizing and training the autoencoder of which the encoder part produces the latent representation. DeepGeni-based classifiers were compared to classifiers trained on three different settings without augmentation: (1) Initial data of 7727 mOTU features without feature selection or latent encoding, (2) Feature selected data (256 mOTU features) without latent encoding, (3) Feature selected data with latent encoding. For each approach, out of three classification algorithms (SVM, RF, and NN), the best-performing one was selected. Also, a state-of-the-art approach that selects differentially abundant mOTU features and applies a random forest classification algorithm was included in the comparison. As an independent validation setting, the most recent study’s data (Peters) was used as test data, and the rest as source data for training classifiers.

**Figure 2:**
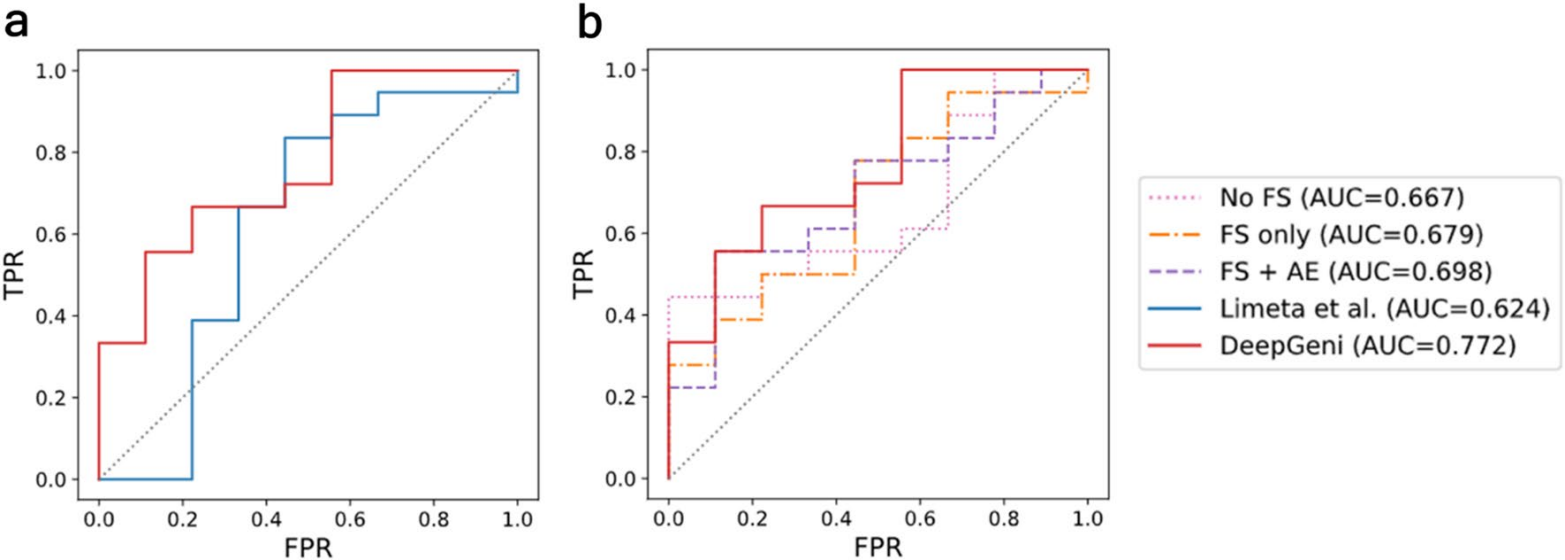
Receiver operating characteristics (ROC) curves of the best classifier for each method predicting ICI response. (**a**) Prediction performance comparison of DeepGeni and the state-of-the-art classifier (Limeta et al.). (**b**) Performance comparison of DeepGeni and alternative approaches using feature selection (FS) and/or autoencoder (AE, FS + AE) without DeepBioGen augmentations.

Remarkably, DeepGeni-based NN classifier surpasses not only the state-of-the-art classifier (Limeta et al.) but the best classifiers of other approaches (Fig. 2). In addition, the rest of DeepGeni-based classifiers (SVM and RF) show better performance than the classifiers of other approaches (Table S2). Also, DeepGeni-based SVM classifier outperforms other classifiers in the cross-study validation setting, displaying the highest generalizability across different studies (Table 2, S3, and S4). The per-study AUC reports in the cross-study validation (Table S4) demonstrate that the DeepGeni-based SVM outperforms other methods on all test datasets except the Matson dataset. However, none of the methods clearly surpass random guessing (AUC = 0.5) on the Matson dataset.

**Table 2:** Averaged AUC in cross-study validation setting.

Approach	No FS			FS only			FS + AE			DeepGeni (FS + DBG + AE)		
Algorithm	SVM	RF	NN	SVM	RF	NN	SVM	RF	NN	SVM	RF	NN
AUC	0.52	0.522	**0.556**	0.564	0.551	**0.585**	**0.602**	0.57	0.598	**0.626**	0.579	0.609
STD	0.156	0.074	0.07	0.107	0.103	0.08	0.06	0.053	0.045	0.209	0.09	0.221

The bolded values indicate the best performance for each approach.

*FS* feature selection, *AE* autoencoder, *DBG* DeepBioGen.

### Key microbiota relevant to ICI response extracted from generalized interpretable autoencoder

**Figure 3:**
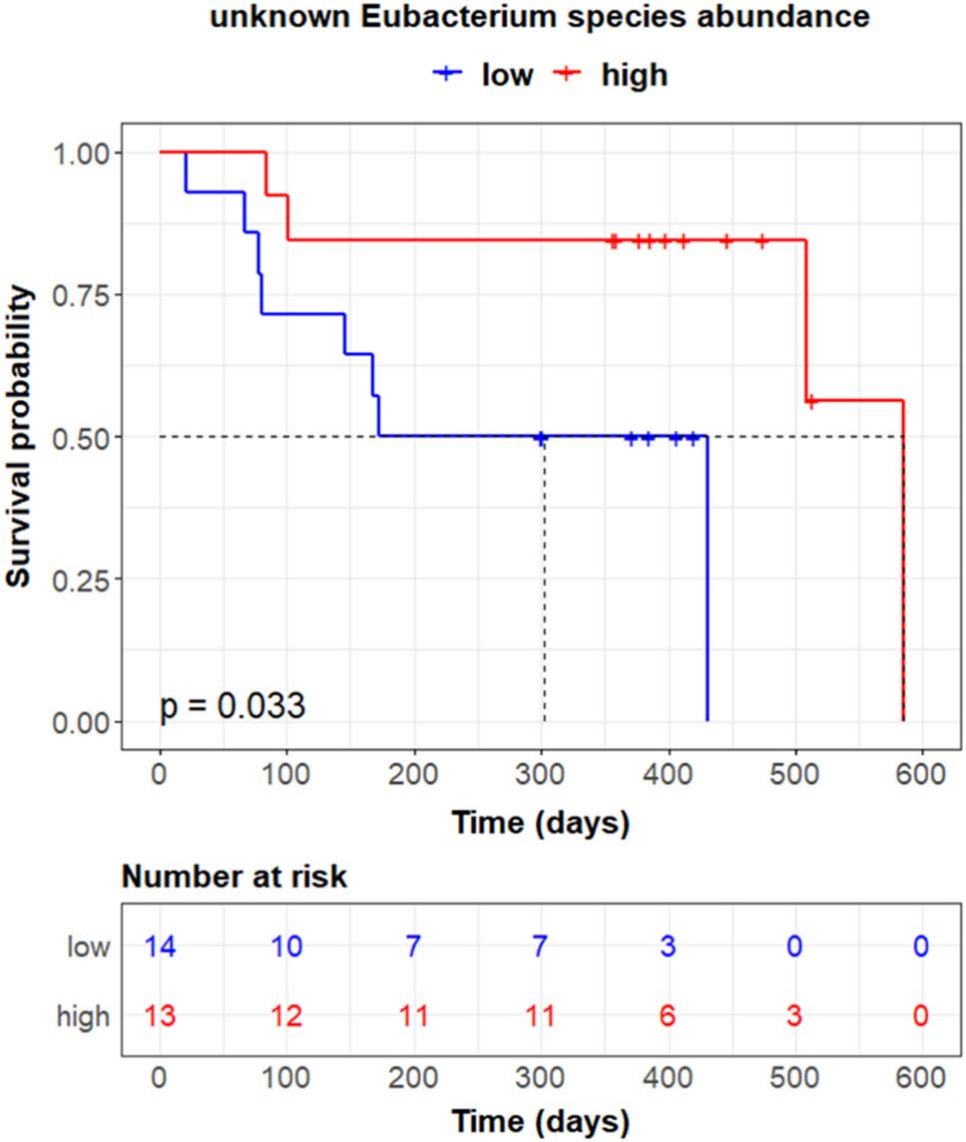
Kaplan–Meier plot of progression-free survival by the relative abundance of unknown Eubacterium species.

The ICI-response-relevant key microbiota was identified by propagating informative signals through the interpretable links from latent variables that play a major role in inducing superior ICI response prediction. The intersection of four sets of microbiota candidates, each of which was derived from a one-study-out setting, resulted in 14 mOTUs (permutation testing *p*-value = 9.0 × 10^–6^). The resulting list categorized into seven families was validated with the literature and statistical tests. The key microbiota identified in the study provide higher resolution in a taxonomic hierarchy and uncover specific species or genera that have not been clarified in the previous studies. Specifically, out of 14, 12 were cross-checked with literature and 11 were specified in lower taxonomic rank (Family to species: 3; Genus to species: 2; Family to genus: 1; Order to family: 5) (Table 3). Interestingly, two ICI-therapy-relevant gut bacteria, *Eggerthella lenta* and unknown *Lactobacillales*, were not reported in previous studies, thus providing new microbe markers for future studies. It is worth noting that the genus Subdoligranulum is closely related to the Faecalibacterium genus. Furthermore, five species, including *Lactobacillus plantarum*, unknown *Ruminococcaceae*, and three unknown *Clostridiales*, displayed statistical significance in differentially abundant testing (unadjusted, Wilcoxon’s rank-sum test). Besides, a high abundance of unknown *Eubacterium* species was significantly associated with prolonged progression-free survival in ICI-treated melanoma patients (Fig. 3).

**Table 3:** The finalist of ICI-response-relevant key microbiota.

mOTU_v2 ID	Consensus taxonomy	Order	Family	Genus	Specified level	Prev level	H-Res	P-val
ref_mOTU_v2_0036	Enterobacteriaceae sp.	Enterobacteriales	Enterobacteriaceae	Escherichia /Shigella	Species	Species^2^	–	
ref_mOTU_v2_0154	Lactobacillus plantarum	Lactobacillales	Lactobacillaceae	Lactobacillus	Species	Family^2^	Yes	*
meta_mOTU_v2_6288	Unknown Lactobacillales		unknown Lactobacillales	Unknown	Family	–	–	
ref_mOTU_v2_0642	Eggerthella lenta	Eggerthellales	Eggerthellaceae	Eggerthella	Species	–	–	
ref_mOTU_v2_0884	Anaerotruncus colihominis	Clostridiales	Ruminococcaceae	Anaerotruncus	Species	Family^1^	Yes	
ref_mOTU_v2_4738	Subdoligranulum sp.			Subdoligranulum	Species	Family^1^	Yes	
ref_mOTU_v2_0281	Ruminococcus lactaris			Ruminococcus	Species	Genus^1,8^	Yes	
meta_mOTU_v2_6557	Unknown Ruminococcaceae			Unknown Ruminococcaceae	Genus	Family^1^	Yes	**
meta_mOTU_v2_6657	Unknown Eubacterium		Eubacteriaceae	Eubacterium	Species	Genus^1,8^	Yes	#
meta_mOTU_v2_5411	Unknown Clostridiales		Unknown Clostridiales	Unknown	Family	Order^1^	Yes	
meta_mOTU_v2_5669	Unknown Clostridiales							*
meta_mOTU_v2_6760	Unknown Clostridiales							*
meta_mOTU_v2_6795	Unknown Clostridiales							*
meta_mOTU_v2_7550	Unknown Clostridiales							

*: *p* < 0.05, Wilcoxon’s rank-sum test on differential abundance; **: *p* < 0.01, Wilcoxon’s rank-sum test; #: *p* < 0.05, log-rank test on progression-free survival distribution difference; H-Res indicates whether the specified taxonomic level is in higher resolution than the previously specified level in other studies.

## Discussion

DeepGeni is a generalized interpretable autoencoder that not only boosts ICI response prediction accuracy in an independent study but provides interpretable links to identify informative taxa contributory to modulating ICI response. The improved generalizability of DeepGeni is supposed to be derived from augmented microbiome data generated by DeepBioGen, a GAN-based data augmentation procedure. We plotted the augmented data along with source and test data in 2-D space using t-SNE algorithm^27^ to understand the potential role of the augmentation (Fig. S1). Interestingly, the augmented data filled gaps between the source and test data in the embedding space, suggesting that the use of augmentation could help overcome the generalization barrier.

The latent representation learned by the generalized autoencoder with the augmented data seems to enable the trained classifiers more resilient to unseen data distributions. Also, DeepGeni extracted microbial species informative to predict ICI response in higher resolution than other studies. The specified species could be a helpful basis for establishing ICI-promoting FMT guidelines to specify donor and donee. Moreover, the identified species may offer a possibility to develop pre or probiotics targeting improved outcomes of ICI therapy.

Landmark studies showed the translational relevance of commensal gut microbiota affecting response to immune checkpoint blockades through clinical cohorts^1–3,8,9^. However, our understanding of how gut microbes might influence ICI response remains lacking, although some studies partially explain potential mechanisms at a high level, such as low diversity and imbalanced microbiota^28–33^. This study suggests specific bacterial taxa derived from the interpretation of deep generative models that brought the best performance in ICI response prediction and the taxa that were not able to be extracted from the available data with traditional statistical methods. The findings could help direct future studies and formulate potential mechanisms of different responses.

Although this study produces the generalized list of ICI-response-relevant key microbial taxa over the available datasets, the ability to statistically validate the identified microbial taxa is bounded by the size of the available data. This could limit the possibility of being validated for some of the key microbial taxa as they were identified by taking advantage of the out-of-distribution augmented data and it may not be eligible to use the augmented data for statistical validation. However, there still remains the possibility of being validated in larger data sets once they become available.

DeepGeni was applied to examine microbiome potentially modulating ICI response in this study but it is highly extensible for identifying microbiome-driven human phenotypes or even for applying other types of biological and ecological data such as genome and metagenome profiles.

## Conclusion

We proposed DeepGeni, a generalized interpretable autoencoder that learns a latent representation of microbiome profiles. The learned representation can improve ICI response prediction on unseen data and suggest the most informative microbial taxa involved in modulating ICI response. In the future study, this work can be extended to other types of features extracted from the microbiome data such as functional-level features that have been shown to exhibit more discriminative powers in certain diseases^34^.

## Supplementary Information

Supplementary Information.

## Data Availability

Gut microbiome datasets analysed during the current study are available in European Nucleotide Archive with the accession numbers PRJEB22893^1^, PRJNA399742^2^, PRJNA397906^9^, and PRJNA541981^22^.

## Abbreviations

ICI: Immune checkpoint inhibitor
FMT: Fecal microbiota transplantation
mOTU: Marker gene-based operational taxonomic unit
GAN: Generative adversarial network
SVM: Support vector machine
RF: Random forest
NN: Feedforward neural network
AUC: Area under the receiver operating characteristics curve
ROC: Receiver operating characteristics
