# Supplementary material for: DeepGeni: deep generalized interpretable autoencoder elucidates gut microbiota for better cancer immunotherapy
Source: Sci Rep. 2023 Mar 21;13:4599. doi: 10.1038/s41598-023-31210-w (PMC10030841; doi:10.1038/s41598-023-31210-w)
Supplement: Supplementary file 1 — Supplementary Information. [file 41598_2023_31210_MOESM1_ESM.pdf]

Supplementary Materials

DeepGeni: Deep generalized interpretable autoencoder elucidates gut microbiota for better cancer immunotherapy

Min Oh[1] and Liqing Zhang[1, *]

[1]Department of Computer Science, Virginia Tech, Blacksburg, VA, USA

[*]Correspondence: lqzhang@cs.vt.edu

Contents

- **Figure S1.** t-SNE visualization of source, augmented, and test data
- **Figure S2.** Overlaps between four sets of 256 features derived from cross-study validation
- **Table S1.** Hyper-parameter grid for optimizing classifiers
- **Table S2.** AUC of the classifiers trained with different approaches
- **Table S3.** Accuracy metrics in cross-study validation setting
- **Table S4.** AUC per fold in cross-study validation setting

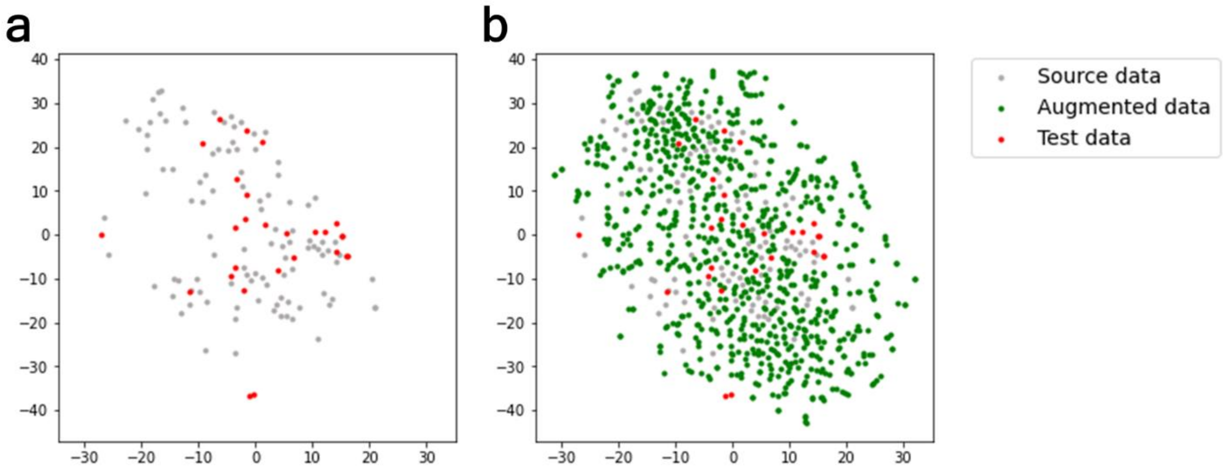

Figure S1. t-SNE visualization of source, augmented, and test data**.** (**a**) Test data is shown in red (Peters dataset) and the source data is shown in gray which is composed of Gopalakrishnan, Matson, and Frankel datasets. (**b**) Augmented data generated by DeepBioGen is plotted in green with the source and test data in (a).

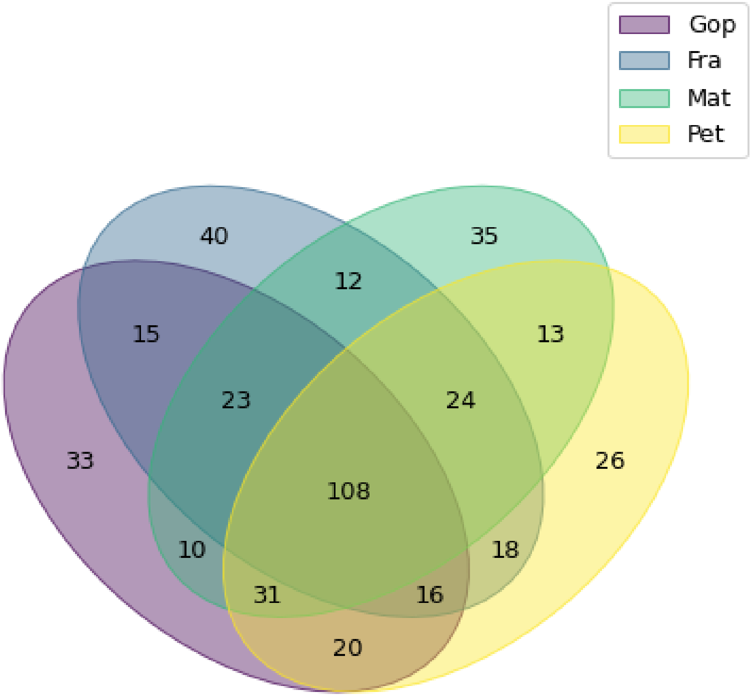

Figure S2. Overlaps between four sets of 256 features derived from cross-study validation. Each feature set is denoted and colored by leave-out study. Gop indicates 256 features derived by using Gopalakrishnan dataset as test data and fitting extremely randomized trees on the rest datasets as training data. Likewise, Mat stands for Matson, Fra for Frankel, and Pet for Peters dataset.

Table S1. Hyper-parameter grid for optimizing classifiers

Classification algorithm	Hyper-parameter	Parameter grid
SVM	Kernel	Linear and radial basis function (RBF)
	Regularization penalty *C*	2^{-4}, 2^{-3}, 2^{-2}, 2^{-1}, 2^{0}, 2^{1}, 2^{2}, and 2^{4}
	Gamma	'Scale' (= 1/(n_features*X.var()) and<br>'Auto' (=1/n_features)
RF	# of estimators	2^{7}, 2^{8}, 2^{9}, and 2^{10}
	Maximum # of features for the best split	Square root and log2 of n_features
	Split criterion	Gini impurity and information gain
NN	Hidden layers (hidden units)	3 layers (128, 64, 32),<br>4 layers (128, 64, 32, 16), and<br>5 layers (128, 64, 32, 16, 8)
	Learning rate	Constant (0.001),<br>invscaling (0.001/ pow(t, power_t) where t is time step), and<br>adaptive (keep learning rate as long as training loss is decreasing, otherwise divide the current learning rate by 5)
	Alpha (L2 penalty)	0.0001, 0.001, 0.01, and 0.1

- SVM: support vector machine; RF: random forest; NN: feedforward neural network

Table S2. AUC of the classifiers trained with different approaches

Approach	Limeta et al.	No FS			FS only			FS + AE			DeepGeni (FS + DBG + AE)		
Algorithm	RF	SVM	RF	NN	SVM	RF	NN	SVM	RF	NN	SVM	RF	NN
AUC	0.624	**0.667**	0.543	0.531	0.673	0.574	**0.679**	**0.698**	0.673	0.605	0.744	0.673	**0.772**

- FS: feature selection; AE: autoencoder; DBG: DeepBioGen

Table S3. Accuracy metrics in cross-study validation setting

Approach	Classifier	AUROC	AUPRC	REC	PRE	F1
FS + DBG + AE	SVM	**0.626**	**0.678**	**0.587**	**0.677**	**0.595**
	RF	0.579	0.612	0.544	0.642	0.561
	NN	0.609	0.652	0.523	0.599	0.548
FS + AE	SVM	0.602	0.643	0.514	0.590	0.522
	RF	0.570	0.611	0.450	0.566	0.482
	NN	0.598	0.636	0.524	0.564	0.532
FS Only	SVM	0.564	0.623	0.524	0.599	0.535
	RF	0.551	0.600	0.389	0.513	0.410
	NN	0.585	0.639	0.453	0.571	0.492
No FS	SVM	0.520	0.608	0.497	0.449	0.406
	RF	0.522	0.615	0.436	0.554	0.440
	NN	0.556	0.625	0.496	0.535	0.500

Table S4. AUC per fold in cross-study validation setting

Approach		No FS			FS only			FS + AE			DeepGeni (FS + DBG + AE)		
Algorithm		SVM	RF	NN	SVM	RF	NN	SVM	RF	NN	SVM	RF	NN
Test data	Gopalakrishnan	0.37	0.617	0.597	0.558	0.682	0.571	0.734	0.649	0.669	**0.779**	0.601	0.747
	Frankel	0.642	0.478	0.626	0.605	0.507	0.605	0.524	0.528	0.584	**0.658**	0.586	0.624
	Matson	0.4	0.5	0.469	0.42	0.4	0.486	0.45	0.4	**0.533**	0.32	0.5	0.292
	Peters	0.667	0.543	0.531	0.673	0.574	0.679	0.698	0.673	0.605	**0.744**	0.673	0.772

- FS: feature selection; AE: autoencoder; DBG: DeepBioGen
